# Kinomic profiling identifies focal adhesion kinase 1 as a therapeutic target in advanced clear cell renal cell carcinoma

**DOI:** 10.18632/oncotarget.16352

**Published:** 2017-03-18

**Authors:** Arindam P. Ghosh, Christopher D. Willey, Joshua C. Anderson, Karim Welaya, Dongquan Chen, Amitkumar Mehta, Pooja Ghatalia, Ankit Madan, Gurudatta Naik, Sunil Sudarshan, Guru Sonpavde

**Affiliations:** ^1^ Department of Radiation Oncology, University of Alabama Birmingham (UAB) Medical Center, Birmingham, Alabama, USA; ^2^ Department of Biostatistics, UAB Medical Center, Birmingham, Alabama, USA; ^3^ Department of Medicine, Section of Hematology-Oncology, UAB Medical Center, Birmingham, Alabama, USA; ^4^ Department of Medicine, UAB Medical Center, Birmingham, Alabama, USA; ^5^ Department of Urology, UAB Medical Center, Birmingham, Alabama, USA; ^6^ Birmingham VA Medical Center, Birmingham, Alabama, USA

**Keywords:** clear cell renal cell carcinoma, kinomics, focal adhesion kinase, metastasis, therapeutic validation

## Abstract

The introduction of targeted therapies has caused a paradigm shift in the treatment of metastatic clear cell (cc)-renal cell carcinoma (RCC). We hypothesized that determining differential kinase activity between primary and metastatic tumor sites may identify critical drivers of progression and relevant therapeutic targets in metastatic disease. Kinomic profiling was performed on primary tumor and metastatic tumor deposits utilizing a peptide substrate microarray to detect relative tyrosine phosphorylation activity. Pharmacologic and genetic loss of function experiments were used to assess the biologic significance of the top scoring kinase on *in vitro* and *in vivo* tumor phenotypes. Kinomics identified 7 peptides with increased tyrosine phosphorylation in metastases that were significantly altered (p<0.005). Based on these peptides, bioinformatics analyses identified several candidate kinases activated in metastases compared to primary tumors. The highest ranked upstream kinase was Focal Adhesion Kinase 1 (FAK1). RCC lines demonstrate evidence of elevated FAK1 activation relative to non-transformed renal epithelial cells. Pharmacologic inhibition of FAK1 with GSK2256098 suppresses *in vitro* tumor phenotypes. In turn, FAK1 knockdown in RCC cells suppresses both *in vitro* phenotypes and *in vivo* tumor growth. Collectively, these data demonstrate functional activation of FAK1 in metastases and provide preclinical rationale for targeting this kinase in the setting of advanced ccRCC.

## INTRODUCTION

Despite multiple targeted agents in the therapeutic armamentarium for clear cell (cc)-renal cell carcinoma (RCC), this disease remains largely incurable. The median overall survival (OS) is approximately 2 to 2.5 years when employing vascular endothelial growth factor (VEGF) inhibitors (e.g. sunitinib, pazopanib, bevacizumab) [1, 2]. Second-line therapy following these primary VEGF inhibitors target Programmed Death (PD)-1 (nivolumab), VEGF (cabozantinib and axitinib) or the combination of VEGF and fibroblast growth factor (FGF) (lenvatinib) plus mTOR (everolimus), and yield median overall survival of 15 to 25 months [3–7]. Thus, the incremental improvement in outcomes provided by these agents may have reached a threshold with further improvements likely requiring the targeting of new molecules. Hence, the discovery of novel therapeutic targets is of enormous importance.

Continuing to investigate the role of novel kinase inhibitors of alternate targets may be reasonable. Indeed, multiple kinases are likely responsible for progression and metastasis and the discovery of novel kinases may offer the opportunity to therapeutically target them with kinase inhibitors [8]. Moreover, kinases are therapeutically actionable and the manufacture of kinase inhibitors is readily accomplished. While large scale expression data sets can be informative, there may be discrepancies between expression and kinase activity. Hence, actionable targets may be better inferred through kinomic approaches. For example, high throughput kinome profiling using flow-through peptide microarrays has identified potential therapeutic targets in pediatric brain tumors [9].

Preliminary studies demonstrate the feasibility of kinomics profiling of primary renal tumors [10]. However, the major morbidity and mortality of this disease is associated with the development of metastasis. Hence, we undertook a study to comprehensively measure the kinomic activity of primary ccRCC relative to metastatic sites as a means to identify novel actionable targets.

## RESULTS

### Phosphotyrosine peptide array profiling of primary and metastatic tumor lysates

Among 96 available fresh frozen ccRCC tumor samples, 92 met quality control criteria for kinase activity. ccRCC tumor was available from 80 primary tumors and 12 metastases (Supplementary Table 1). The percent cellular viability and necrosis were acceptable. Seven peptide probes demonstrated significantly increased tyrosine phosphorylation by protein lysates from metastases relative to protein lysates derived from primary tumor (Table 1). The peptide with the highest phosphorylation by metastatic tumor lysates is derived from FGF receptor (FGFR)-1 with the phosphorylation site corresponding to tyrosine residue 766 (Y766). There are 7 autophosphorylation sites in FGFR1, Y463 (juxtamembrane), Y583/Y585 (kinase insert), Y653/Y654 (the activation loop), Y730 (kinase domain) and Y766 (C-terminal tail) [19]. Phosphorylated tyrosine residues function as docking sites for various adaptor proteins [20]. Some of the adaptor proteins are phosphorylated directly by FGFR [20]. For example, upon phosphorylation the C-terminal Y766 binds PLCγ [19] and Shb [21], which leads to recruitment of FRS2. The binding of the docking proteins to FGFRs leads to activation of multiple signal transduction pathways, including the four main downstream pathways, Ras–Raf–MapK, PI3K–Akt, Stats, and PLCγ [21]. Recent studies indicate that FGFR1 may be a target for advanced RCC [22]. The next most phosphorylated substrate is a peptide derived from FAK1. FAK is phosphorylated in response to integrin engagement, mitogenic neuropeptides, lysophosphatidic acid, platelet-derived growth factor, activated Rho, and selected oncogenes leading to the formation of docking sites for a variety of signaling molecules that determine cell morphology, locomotion, proliferation, differentiation, and apoptosis [23–25]. Several sites of tyrosine phosphorylation have been identified in FAK which serve to modulate FAK kinase activity or mediate FAK interaction with SH2-domain containing proteins, including the major autophosphorylation site Y397 essential for the majority of FAK functions [23, 26]. FAK auto-phosphorylation at Y397 leads to binding of Src-family kinases to the phosphorylated site and subsequent Src-mediated phosphorylation of the FAK kinase domain activation loop (Y576/577 culminating in the formation of an activated FAK-Src complex [23, 24].

**Table 1:** Comparative analysis of metastatic and primary tumor utilizing phosphotyrosine peptide substrate array

Peptide ID	Sequence	Uniprot	P value	Fold change(Metastasis vs. primary)
FGFR1_761_773	TSNQEYLDLSMPL	P11362	0.0048	1.645723343
FAK1_569_581	RYMEDSTYYKASK	Q05397	0.00475	1.552727699
ACHD_383_395	YISKAEEYFLLKS	Q07001	0.00488	1.504278183
K2C8_425_437	SAYGGLTSPGLSY	P05787	0.0046	1.490841389
EGFR_1118_1130	APSRDPHYQDPHS	P00533	0.002	1.470849514
EPHB4_583_595	IGHGTKVYIDPFT	P54760	0.000609	1.356128693
MBP_259_271	FGYGGRASDYKSA	P02686	0.00456	1.205086231

Metastasis and primary tumor protein lysates were subjected to kinomic analysis utilizing a phosphotyrosine (PTK) peptide substrate array. 7 PTK peptides were identified as having statistically significant higher levels of phosphorylation upon treatment with metastatic tumor protein lysate relative to primary tumor.

**Figure 1:**
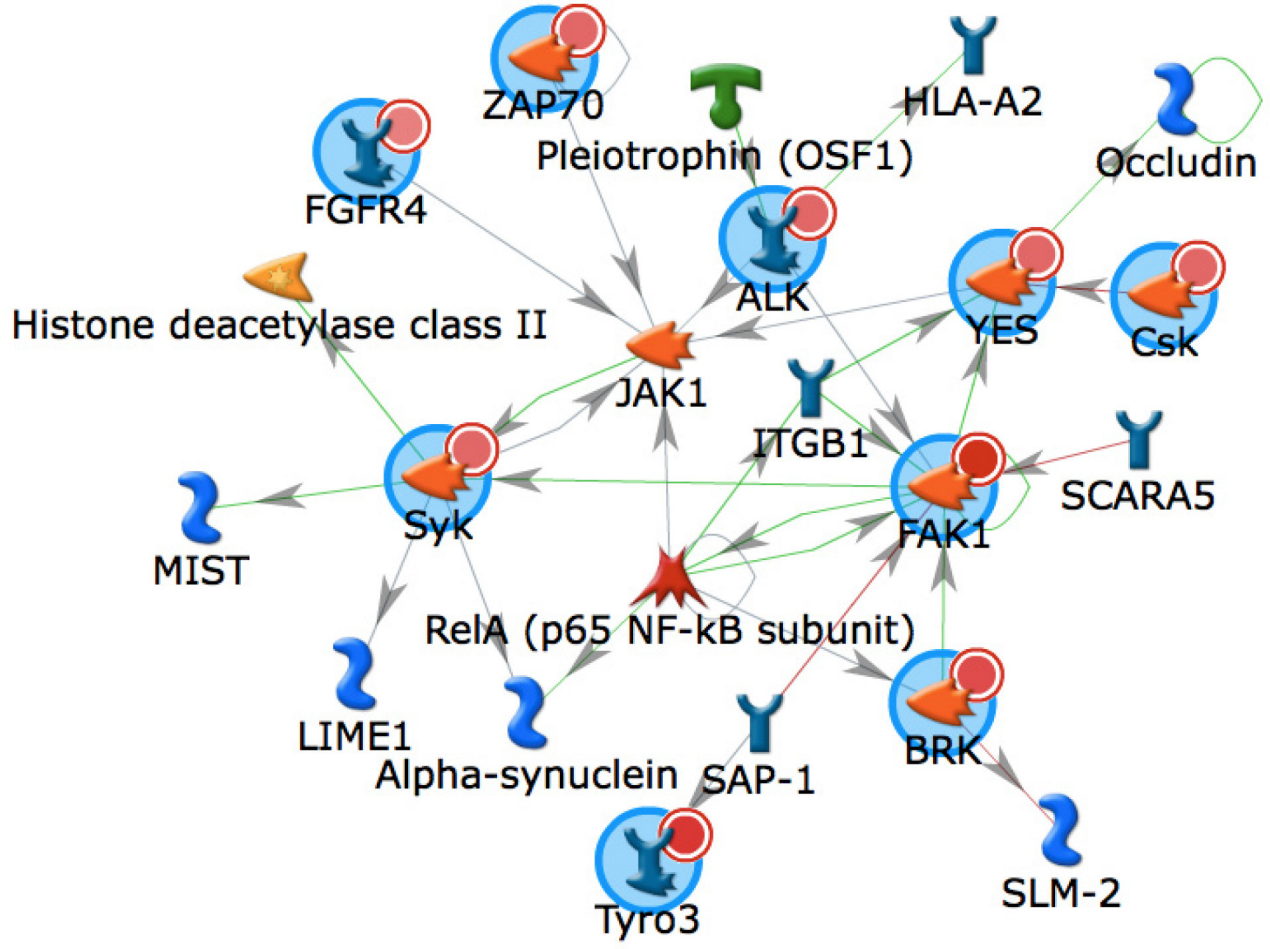
Biological network of kinases identified as activated in metastatic RCC A network generated using GeneGo MetaCore of kinases output from the UpKin PamApp(v8.0) in BioNavigator (v6.2). Kinases identified as activated in metastatic RCC were uploaded by uniprot ID and the AutoExpand network was used with a maximum network size of 25 nodes, with orphaned nodes (non-interconnected) excluded. Canonical pathways were deselected. Input kinases are denoted with a small red circle within a larger blue circle. Direction of literature-annotated interactions are indicated by arrowheads allowing interconnecting lines, with green lines indicating positive, red lines indicating negative, and gray lines indicating complex interactions.

We next performed algorithmic analyses of these 7 phosphopeptides as a means to infer candidate upstream kinase activated in metastasis relative to primary RCC (Table 2 and Supplementary File). This analysis identified kinases implicated in advanced renal cancer including AXL, ALK1, and MET. Several recent studies, both preclinical and clinical, demonstrate that some of these kinases are actionable targets in metastatic RCC [27, 28]. The top scoring kinase was FAK1 (Table 2 and Supplementary File). In addition, PYK2, also referred to as FAK2, also was identified as an activated kinase in metastasis. While FAK1 activation is associated with FAK1 autophosphorylation at Y397 and subsequent FAK1 phosphorylation at residues Y576 and Y577, it is also implicated in the phosphorylation of several other phosphopetides identified in the array demonstrated by the high Kinexus score and Hit % (Table 2 and Supplementary File). A network map of kinases predicted to be activated in metastatic RCC is shown in Figure 1. Collectively, these data indicate activation of FAK1 signaling in renal cancer metastasis. In turn, these data provide rationale for exploring the role of FAK1 in renal carcinogenesis and as potential target for RCC.

**Table 2:** Upstream kinase prediction based on phosphotyrosine peptide substrate array

Kinexus Kinase	Uniprot	Kinexus Score	Hit %
**FAK1**	Q05397	3979	50
**TYRO3**	Q06418	2421	40
**AXL**	P30530	2428	35
**BRK**	Q13882	3205	35
**ALK**	Q9UM73	1038	30
**CSK**	P41240	1794	30
**MER (MERTK)**	Q12866	1941	30
**PYK2 (PTK2B)**	Q14289	2440	30
**SYK**	P43405	2292	30
**YES1**	P07947	2658	30
**ZAP70**	P43403	1918	30
**ARG (ABL2)**	P42684	2030	25
**CTK (MATK)**	P42679	2191	25
**FGFR4**	P22455	1759	25
**FRK**	P42685	1526	25
**MET (HGF Receptor)**	P08581	1825	25
**SRM (SRMS)**	Q9H3Y6	1655	25

Algorithmic analyses of the 7 PTK peptides found to be significantly different was performed to identify candidate upstream kinases activated in metastasis relative to primary tumor. Kinase score and Hit% were generated as described in the methods.

### Basal phosphorylation of FAK1 in RCC lines and effects of pharmacologic FAK1 inhibitor on *in vitro* phenotypes

**Figure 2:**
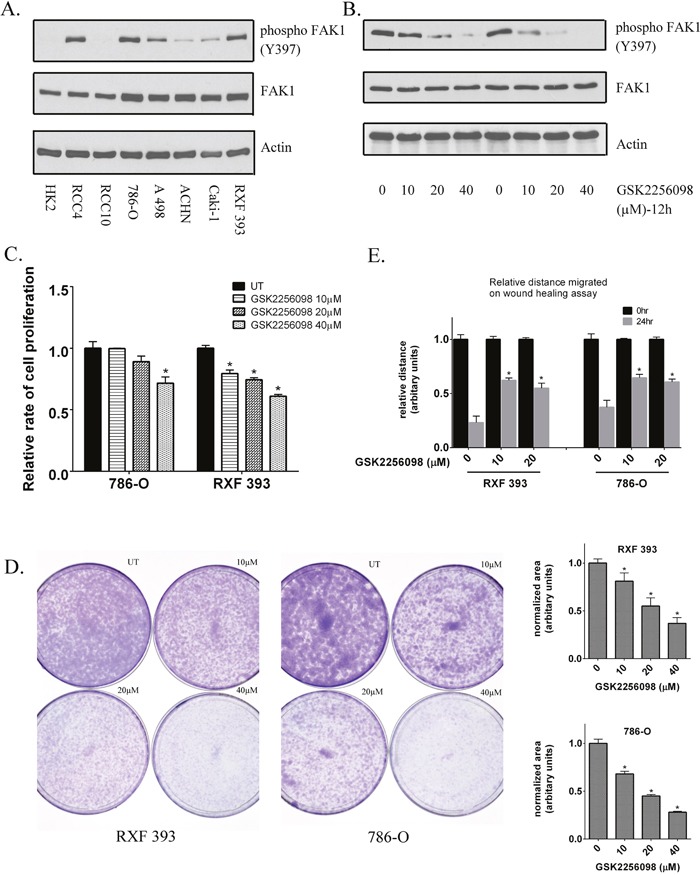
Inhibition of FAK kinase activity in RCC lines **(A)** Levels of total and phosphorylated FAK1(Y397) were assessed in RCC lines compared to untransformed HK2 renal epithelial cells. Actin was used as a loading control. **(B)** Inhibition of FAK1 phosphorylation (Y397) by GSK2256098 was assessed in 786-O and RXF393 cells by immunoblot analysis at 12 hours post-treatment. Data is representative of 3 independent experiments. **(C)** Proliferation was measured in RXF393 and 786-O cells at 48 hours post-treatment in the presence or absence of GSK 2256098. Data was quantified from 3 independent experiments. * p<0.05 and error bars represent SEM. **(D)** Colony formation assay in RXF393 and 786-O cells in the presence or absence of GSK2256098. Data is representative of 3 independent experiments. * p<0.05 and error bars represent SEM. **(E)** Migration in 786-O and RXF 393 cells measured by wound healing assay at 24 hours post-treatment with GSK2256098. Data is representative of 3 independent experiments. * p<0.05 and error bars represent SEM.

Based on these data, we next determined the relevance of FAK1 as a target in ccRCC. We first analyzed FAK1 expression in a panel of renal epithelial lines which included HK2 cells (immortalized, non-transformed proximal tubular epithelial cells) and RCC cells (Figure 2A). All lines tested expressed FAK1 at the protein levels. In, contrast, most RCC lines demonstrated higher levels of phosphorylated FAK1(Y397) in comparison to HK2 cells (Figure 2A). As noted, Y397 is an autocatalytic site of FAK1 and phosphorylation at this residue is associated with higher FAK1 kinase activity [23, 26]. To better assess the biologic relevance of FAK1, we next assessed the effects of FAK1 inhibition in 786-O and RXF-393 cells which demonstrate relatively high levels of FAK1 activation. Our initial studies focused on pharmacologic FAK1 inhibition with the use of the agent GSK2256098, a highly selective inhibitor of FAK1. Treatment of 786-O and RXF-393 cells with GSK2256098 resulted in a dose-dependent decrease in Y397 phosphorylation without effects on total FAK1 levels (Figure 2B).

We measured the change in the proliferative rates of RCC cells in response to GSK2256098. 786-O and RXF393 cells treated with GSK2256098 demonstrated reduced proliferation relative to untreated cells (Figure 2C). To assess the effect of inhibiting FAK kinase activity on the clonogenic potential of these cells, we performed a colony formation assay in 786-O and RXF393 cells in the presence of increasing concentrations of GSK2256098 and compared the results to vehicle treated cells. GSK2256098 treatment resulted in a concentration-dependent decrease in colony formation (Figure 2D). FAK1 is known to participate in cell-cell communication and adhesion [23–25]. We therefore tested the effects on GSK2256098 on RCC migration via wound healing assay as previously described [29]. Inhibition of FAK kinase activity by GSK2256098 decreased wound healing in both 786-O and RXF-393 cells (Figure 2E and Supplementary Figure 1). Importantly, FAK1 inhibition reduced wound healing at relatively low doses of GSK2256098. In 786-O cells, FAK1 inhibitor reduced wound healing at doses that did not impact cellular proliferation indicating that the effects on cell migration could not be attributed to reduced cell number.

### Knockdown of FAK1 in RCC cells recapitulates effects of pharmacologic inhibition

**Figure 3:**
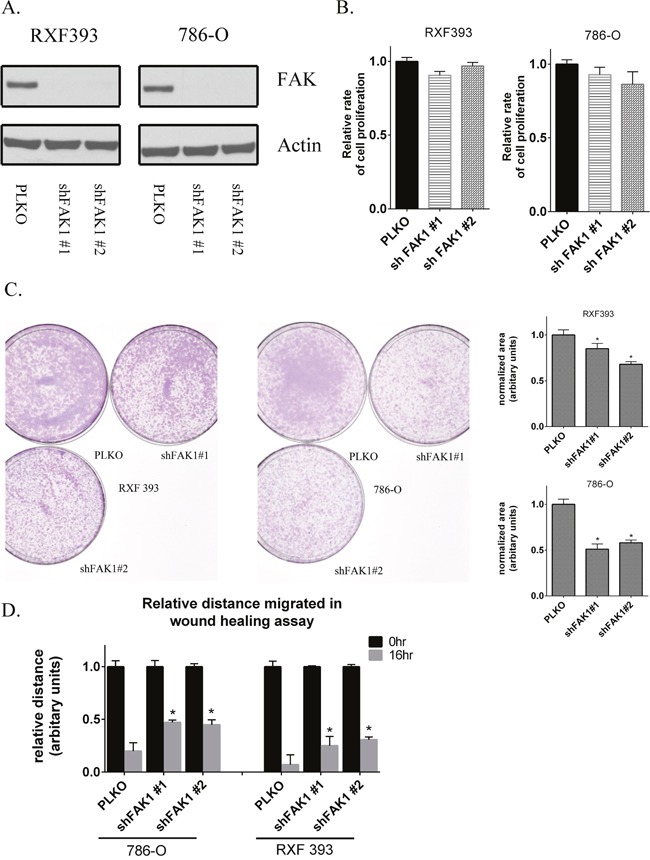
Genetic knockdown of FAK1 in RCC lines recapitulates the effects of FAK1 kinase inhibitor **(A)** 786-O and RXF393 FAK1 knockdown stable cells were generated by lentiviral transduction. Two different shRNA constructs targeting different regions of FAK1 gene were used. Level of FAK protein was assessed by immunoblot analysis and compared to Actin loading controls. **(B)** Proliferation of cells measured by using Cell Titer Glo™ in FAK1 knockdown RXF393 and 786-O cells in comparison to PLKO controls 48 hours after seeding the cells in 96 well plates. Data is representative of 3 independent experiments. **(C)** Clonogenic ability of cells with stable knockdown of FAK1 was measured in comparison to PLKO control transduced cells. Data is representative of 3 independent experiments. **(D)** Migration in 786-O and RXF 393 cells either transduced with PLKO control or shRNA against FAK1 was measured by wound healing assay at 16 hours. Data is representative of 3 independent experiments. * p<0.05 and error bars represent SEM.

Given the effects of FAK1 pharmacologic inhibition on *in vitro* phenotypes, we wanted to validate the effects of FAK1 inhibition via genetic loss of function experiments. We used lentivirus to stably knockdown FAK1 expression via shRNA. Immunoblotting after puromycin selection demonstrated reduced FAK1 protein expression in cells transduced with two non-overlapping shRNA constructs relative to control vector (PLKO) transduced cells (Figure 3A). FAK1 knockdown cells did not demonstrate significant effects on cellular proliferation (Figure 3B) at 48 hours. In contrast, both FAK1 knockdown clones demonstrated reduced colony formation relative to control vector cells (Figure 3C). In addition, FAK1 knockdown in RCC cells reduced wound healing (Figure 3D and Supplementary Figure 2). Collectively, these results are in agreement with the effects of the FAK kinase inhibitor GSK2256098 in RCC lines.

### FAK1 knockdown suppresses RCC growth *in vivo*

**Figure 4:**
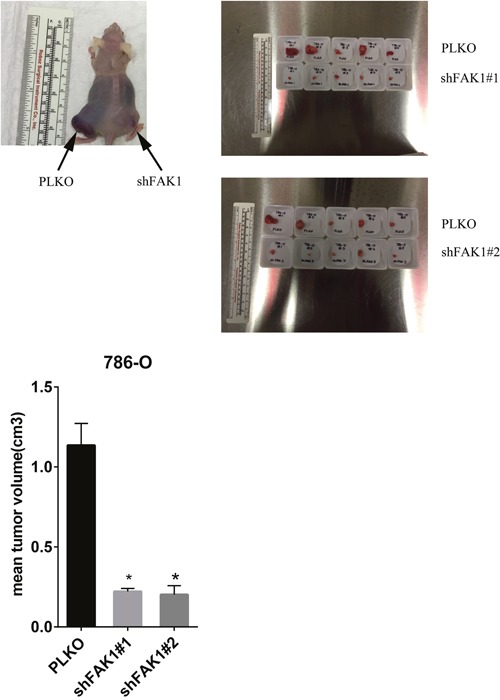
Ablation of FAK1 in 786-O cells decreases tumor growth in mouse xenografts 786-O cells stably transduced with control vector or FAK1 shRNA were injected into the flanks of athymic nude mice. 5 animals were injected per group and tumors harvested at 8 weeks and measured. *p< 0.05 relative to PLKO controls.

Based on the data from the *in vitro* studies, we next determined the *in vivo* effects of FAK1 inhibition in RCC cells. 786-O cells stably transduced with control vector and FAK1 shRNA were analyzed via subcutaneous xenograft assay in nude mice (Figure 4 and Supplementary Figure 3). Tumor cells transduced with 2 different shRNA constructs targeting FAK1 in 786-O cells showed a marked reduction in tumor growth in comparison to control cells. Thus our xenograft studies validate the results observed from FAK1 inhibition *in vitro* and suggest that FAK1 may be a therapeutic target in ccRCC.

## DISCUSSION

This study is the first analysis of comprehensive kinomic profiling comparing primary with metastatic ccRCC tumor. The kinomics platform employed measures the actual functional activity (i.e. ability to phosphorylate a tyrosine residue) of tyrosine kinases on peptide substrates which is likely more physiologically relevant than kinase expression [30]. We studied 80 primary and 12 metastatic ccRCC tumors and identified 7 peptide substrates with significantly increased tyrosine phosphorylation by metastatic samples relative to primary tumor samples including FGFR1, FAK1, ACHD, K2C8, EGFR, EPHB4 and MBP. It may be reasonable to hypothesize that kinases upstream of these substrates mediate progression to metastatic disease, and therefore therapeutic targets across a broad population of unselected patients with metastatic ccRCC.

When examining the panel of increased substrate phosphorylation in metastases in the overall population, FGFR1 demonstrated the highest fold difference compared to primary tumors. In addition to VEGF and PDGF, the fibroblast growth factor (FGF) pathway mediates angiogenesis and may be especially critical in mediating resistance to VEGF inhibitors [31–33]. Notably, lenvatinib, a potent small molecule kinase inhibitor of all FGF and VEGF receptors, has extended progression-free survival in combination with everolimus following prior VEGF inhibitors for metastatic RCC. More selective FGF inhibitors may also warrant investigation to improve the therapeutic index.

The eligibility of the other activated kinases as therapeutic targets in ccRCC is not as well understood although preclinical studies demonstrate the potential of candidate kinase identified in the current study including AXL, ALK1, and MET. FAK1 is known to be a linker between extracellular signals transmitted through integrins and growth factor receptors and is associated with invasion and metastasis in other solid malignancies [34, 35]. Indeed, FAK1 was the highest ranked upstream kinase based on combined scoring criteria (Table 2). Hence, we targeted the preclinical inhibition of FAK1 for further investigation. Indeed, FAK1 inhibition using GSK2256098 suppressed *in vitro* ccRCC cell line proliferation and FAK1 knockdown in ccRCC cells suppressed both *in vitro* and *in vivo* tumor growth. Preclinical data exist to support the induction of anoikis in RCC by targeting FAK survival signaling by quinazoline compounds [36]. However, FAK1 inhibitors have not been investigated in clinical trials enrolling specifically ccRCC patients, although phase I clinical trials are ongoing in solid tumors. Interestingly, in one phase I trial investigating a FAK1 inhibitor, the agent was tolerable and one of 3 patients with heavily pretreated RCC demonstrated durable stability [37]. In addition to suggesting the potential importance of FGFR1 and FAK1 inhibitors in metastatic disease, these agents may also warrant investigation as adjuvant therapy following surgical resection of localized high-risk disease to prevent metastasis. In this regard, more specific inhibitors may be preferable, given the poor tolerability of multi-targeted tyrosine kinase inhibitors in trials of adjuvant therapy [38]. K2C8, whose peptide substrate is predicted to be downstream of FAK1 and FGFR1-4 (http://www.phosphonet.ca/kinasepredictor.aspx?uni=P05787&ps=Y437), may be a component of signaling from estrogen receptors and AKT1, while EPHB4 (peptide substrate that is downstream of BRK, ARG, YES1 and SYK) (http://www.phosphonet.ca/kinasepredictor.aspx?uni=P54760&ps=Y590) may play a role in angiogenesis [39, 40]. Conversely, clinical trials evaluating EGFR inhibitors have demonstrated marginal activity in both ccRCC and non-ccRCC [41–43]. Interestingly, VEGFR2 and PDGFR, which are inhibited by all of the currently approved TKIs, were not amplified in metastases in the current study. Hence, it is possible that the VEGF and PDGF pathway is more critical for primary tumor progression than for invasion and metastasis.

One limitation of the current study is that the number of metastatic tumor samples eligible for analysis was small. In addition, tumor heterogeneity remains a formidable problem to address, and relevant kinases may not have been identified due to the study of a single tissue sample per tumor. However, the impact of tumor heterogeneity on kinase activity is unclear. Alternative designs for therapeutic target discovery may have been employed, e.g. kinomics in localized tumors associated with clinical recurrence and progression to metastatic disease may also have been examined as potential drivers of disease warranting therapeutic targeting. Indeed, we are currently collecting clinical outcome data to determine their association with kinomic profiles. However, in the current study, we intended to directly identify relevant kinases in metastatic tissue and compare their activity levels with primary renal tumor tissue given that adverse outcomes in patients with ccRCC is primarily in the setting of metastatic disease. The anti-tumor activity of FAK1 inhibition observed in our study may be characterized as modest. However, it is possible that FAK1 inhibition requires prolonged administration to optimize its use. We allowed any level of clear cell component, but the impact of the proportion of clear cell component on clinical behavior and biology is unclear. In addition, the activated kinases and altered signaling pathways identified in metastases compared to primaries may not necessarily constitute therapeutic targets, and may merely be passenger alterations associated with other key unrecognized driver alterations. Nevertheless, our study analyzed metastatic tumors directly and compared their profiles with those of primary kidney tumors, i.e. the kinases were not merely activated in primary tumors and associated with future metastatic progression. Notably, previous molecular profiling of metastatic ccRCC tumor tissue has not been reported from large datasets.

Despite the caveats, our study uses a platform that measures enzymatically active kinases in fresh frozen RCC with clear cell components and identifies some kinases over-active in metastatic tumors that are known to drive tumor growth. In particular, FGFR1 and FAK1 warrant special attention, given that inhibition of these kinases has already demonstrated preclinical or clinical activity in RCC or other malignancies. Our data warrant external validation and proof-of-concept preclinical and clinical trials evaluating relatively specific inhibitors of these kinases. However, a recent phoshoproteomics approach identified evidence of FAK activation in RCC [44]. In addition, future studies should be integrated with genomics data to assess the effects of mutations of genes commonly mutated in ccRCC (e.g. *VHL, SETD2, BAP1*) on the cancer kinome. However, it is also likely that a substantial amount of heterogeneity and stochastic drivers of metastases will be identified as we move forward. Nevertheless, the integration of such data could lead to more rationale and personalized approaches for patients affected by advanced ccRCC.

To conclude, our preclinical data demonstrate the high activity of FAK1 in metastatic ccRCC tissue compared to primary tumor tissue coupled with anti-tumor activity of FAK1 inhibition. Further clinical validation of these data is warranted, potentially as an adjunct in combination with other active agents for metastatic disease or as adjuvant therapy following resection of localized disease.

## MATERIALS AND METHODS

### Clear cell renal cell carcinoma tumors

Fresh frozen ccRCC samples were provided by the Southern Division of the Cooperative Human Tissue Network (CHTN, http://www.chtn.nci.nih.gov) based at the University of Alabama at Birmingham (UAB). All tumor specimens had some component of clear cell histology (ccRCC), the most common histology of kidney cancer. Tissue was snap-frozen or frozen in cryo-embedding media such as OCT^®^ and stored in liquid nitrogen or at −80°C. All specimens were subject to an immediate gross examination by a pathologist. The diagnosis was then verified through frozen section, touch preparations, or subsequent evaluation of permanent histopathology. The CHTN has prepared and follows a set of guidelines and procedures for handling human tissues [11]. An IRB approved protocol at UAB permitted the conduct of this study.

### Kinase activity profiling

Sections of tissue were inspected, and 3-5mm^3^ excisions were made from the main biopsy selecting areas with minimal necrosis or fibrosis. When tissue was grossly heterogenous, larger pieces were taken for lysis to compensate for the potential of kinase-activity heterogeneity. Kinomic profiling of tumor lysates was performed using the PamStation^®^12 high-content phospho-peptide substrate microarray system (PamGene International, Den Bosch, The Netherlands) within the UAB Kinome Core as previously described [9, 12–14]. Briefly, the protein tyrosine kinome (PTK) PamChips^®^ were used to measure global kinase activity. Tumor tissue lysates from the RCC samples (both primary tumor and metastatic tumor) were prepared using M-Per lysis buffer with protease- and phosphatase-inhibitor cocktails (Pierce). The PamChips^®^ were blocked in 2% Bovine Serum Albumin (BSA). Following protein concentration determination (by BCA assay), 6 μg protein was loaded per well of the PamChip^®^ along with standard kinase buffer (supplied by PamGene) containing 100 μM ATP and FITC-labeled anti-phospho-tyrosine antibodies. The assay mix is pumped through the PamChips^®^ with a kinetic image capture program (Evolve software, PamGene) in which exposures of phosphorylated peptide substrates are taken as frequently as every 6 seconds for the length of the program (60 minutes). Signal intensity minus background was taken for each spot after verifying gridding during quality control evaluation. Kinase activity can vary based on collection and tissue procedures, and there are no validated kinase activity ‘housekeeping’ phosphoproteins to correct for global, or non-experimental variable-based changes. Towards this end all samples were batch processed for lysis for this experiment to minimize processing variability, however it is not possible to correct for potential primary collection and storage variation. Samples with poor signal, or array errors were removed prior to analysis as per PamGene standard protocol. This raw data was then analyzed as described below in *Statistical Methods*.

### RCC cell lines

All lines were acquired from ATCC except RXF-393 (NCI) and RCC4 (P. Ratcliffe, Oxford). Cell lines were periodically tested for mycoplasma. No other authentication was performed. Cells were grown in DMEM or RPMI-1640 media supplemented with 10% heat-inactivated fetal bovine serum at 37°C in a humidified 5% CO_2_ atmosphere.

### Reagents and chemicals

RCC cells were treated with increasing concentrations of GSK2256098 (GlaxoSmithKline), a small molecule FAK1 inhibitor, for variable lengths of time to determine toxic concentrations of the drug. For all experiments RCC cells were seeded and treated for 12-24 hrs.

### Colony forming assay

Cells were counted and plated at 1,500 cells in 100mm dishes (in duplicate) and incubated at 37°C, 5% CO_2_ for 10-14 days. Colonies were fixed with 10% (v/v) methanol for 15 min and stained with Giemsa (Sigma) for 20 min for colony visualization. The number of colonies was counted and analyzed from 3 independent experiments using Image J software.

### *In vitro* proliferation assays

Six replicates each of 3,000 cells were seeded into 96-well plates for each experiment and assayed using Cell titer Glo® Luminescent Cell Viability Assay (Promega) according to the manufacturer's protocol. At least 3 independent experiments were performed at three different times and the average data from the 3 independent experiments was quantified using GraphPad Prism software.

### Wound healing assay

Wound healing assays were modified from a protocol previously described [15].786-O and RXF-393 cells were seeded in 6 well plates to achieve about 90% confluence at the time of experiments. Thereafter, the cells were scratched with a 1000-μl pipette tip. Plates were washed twice with PBS in order to remove the detached cells, and incubated using the complete growth medium. The point where the scratch was made was marked and images were taken at 0 hours. Wound closure was measured and images were taken after 12 or 16 hours. The distance migrated was calculated from 3 independent experiments using Image J for analysis. The data was quantified and represented using GraphPad Prism software.

### Cell lysis and immunoblotting

Cells were rinsed once with ice-cold PBS and lysed in ice-cold RIPA buffer (25mM Tris-HCl pH 7.6, 150mM NaCl, 1% NP-40, 1% sodium deoxycholate, 0.1% SDS) supplemented with protease and phosphatase inhibitors. The soluble fractions of cell lysates were isolated by centrifugation at 13,000 rpm for 10 minutes by centrifugation in a microfuge. Protein concentrations were determined using a BCA assay. All immunoblot analyses were performed as previously described [16]. Antibodies were obtained from the following sources: phospho-FAK1 (Y397) and total FAK1 (Invitrogen); Actin (Sigma).

### Generation of stable cell lines

Validated Lentiviral shRNA (FAK1) constructs were purchased from Sigma [TRCN0000196310(CCGGGATGTTGGTTTAAAGCGATTTCTCGAGAAATCGCTTTAAACCAACATCTTTTTTG) and TRCN0000194984(CCGGCAACAGGTGAAGAGCGATTATCTCGAGATAATCGCTCTTCACCTGTTGTTTTTTG)]. shRNAs were co-transfected into 293T cells together with packaging plasmids by following the manufacturer's protocol (Invitrogen ViraPower™ Lentiviral Expression Systems kit, Carlsbad, CA). RXF 393 and 786-O cells were passaged and plated in a 6-well plate and allowed to adhere for 24 h before infection. RXF 393 and 786-O cells were transduced in the presence of polybrene overnight. After 24 h cells were selected by treating with media containing 2 μg/ml puromycin.

### Assessment of *in vivo* tumorigenecity using xenografts

786-O with PLKO.1 empty vector or FAK1 shRNA, were grown and maintained in complete media containing puromycin. Two million cells were collected and resuspended in 150μL of media and mixed with an equal volume of BD Matrigel™ Basement Membrane Matrix (BD Biosciences). These cells were injected into the flanks of athymic nude mice (NU(NCr)-*Foxn1*^nu^ ; Charles River Laboratories) following the Institutional Animal Care and Use Committee (IACUC) protocols at UAB. Five animals were injected per group and assessed over time for the development of tumors. Animals were sacrificed at 8 weeks post-injection and tumors were harvested from the flanks.

### Statistical methods

The degree of phosphorylation on each PamChip peptide probe was measured kinetically using Evolve software (PamGene), that measured FITC labeled anti-phosphotyrosine antibody binding to each phosphorylated peptide substrate during the 60 min assay and were further analyzed using BioNavigator software (PamGene), the open source microarray statistical package, R (www.r-project.org), and the commercial MetaCore (GeneGo, Inc., a Thomson-Reuters Company) knowledge base, to develop pathway maps and biological networks. Peptides with increased phosphorylation were queried based on their phosphorylatable residues (up to 6 per peptide) on www.phosphonet.ca (Kinexus). Two scoring algorithms, ‘V2′ and ‘Proximity’, were used to identify putative upstream kinases responsible for peptide phosphorylation. The ‘score’ is the combination of both V2 and Proximity scores as previously described [17, 18]. In addition, a hit% was also generated as previously described [17, 18]. For each algorithm, the top 10 candidate upstream kinases were identified and combined to a total of 20 candidate upstream kinases for each differentially phosphorylated peptide. The percent hit rate (% hit) (occurrence divided by the number of residues with kinase information) of a kinase within this top 20 list for each phosphopeptide was determined as previously described [17, 18]. All peptides in the metastatic and primary renal tumors were compared using an unpaired students t-test with p<0.005 deemed to be significant. Peptides were uploaded to MetaCore as source-protein Uniprot ID's and a Djikstra's shortest path networking algorithm with 2 steps maximum between nodes was used to generate a network model.

## SUPPLEMENTARY MATERIALS FIGURES, TABLES AND DATA




