# Supplementary material for: Kinomic profiling identifies focal adhesion kinase 1 as a therapeutic target in advanced clear cell renal cell carcinoma
Source: Oncotarget. 2017 Mar 18;8(17):29220–32. doi: 10.18632/oncotarget.16352 (PMC5438725; doi:10.18632/oncotarget.16352)
Supplement: Supplementary file 1 [file oncotarget-08-29220-s001.pdf]

Kinomic profiling identifies focal adhesion kinase 1 as a therapeutic target in advanced clear cell renal cell carcinoma

SUPPLEMENTARY MATERIALS

SUPPLEMENTARY DATA

Supplementary File 1: Kinomic Signal Data

Kinomic read data by cycle for each lysate. Values in ‘All Cycle’ sheet are the signal intensity(brightness) minus local background for each Barcode(row 2)/Array(row 3). Values are sorted by sample (row 6) with increasing cycle number (row 4; pumping steps over time) and then by exposure time (row 5). With phosphorylation of target peptides (PamGene peptide ID [col A], peptide sequence [col B], phosphorylatable residues[C]) over time these values increase. Sheet ‘Transformed-PTK’ includes slope values of postwash (cycle 94) across 10-200ms exposure times that are multiplied by 100, and Log2 transformed for each samples. All values are generated by BioNavigator. Sheet ‘MetPrimMeans’ includes the experimental means per group, and the BioNavigator generated unpaired p value for each peptide.

See Supplementary File 1

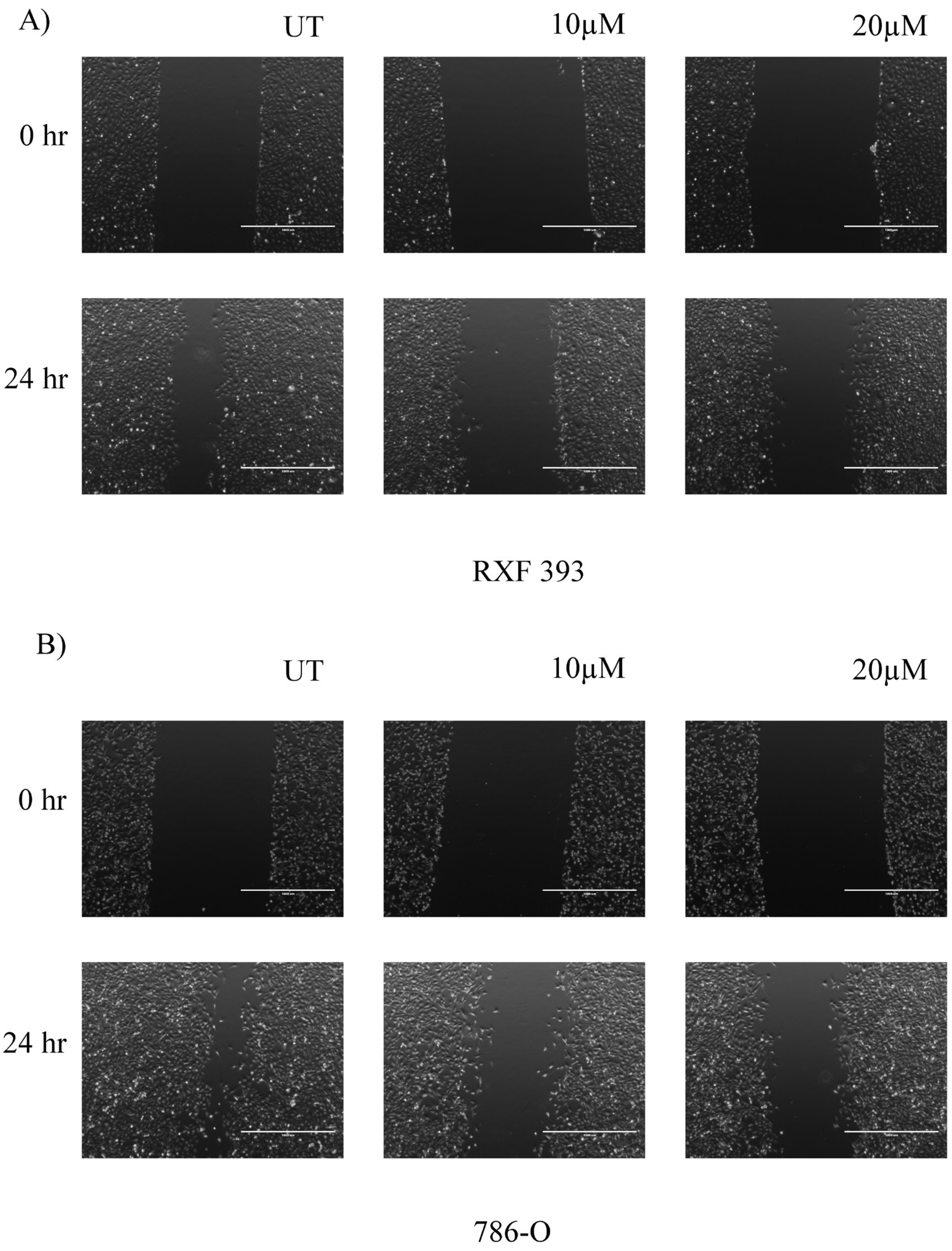

Supplementary Figure 1: FAK1 kinase inhibition decreases migration of RXF393 and 786-O RCC cell lines. **(A)** Migration was assessed in RXF 393 cells in the absence or presence of GSK2256098 using a wound healing assay and the relative distance of migration was measured at 24 h in 3 replicate experiments and quantified. Representative images are shown. **(B)** Migration was assessed in 786-O cells in the absence or presence of GSK2256098 using a wound healing assay and the relative distance of migration was measured. Representative images from 3 replicate experiments are shown.

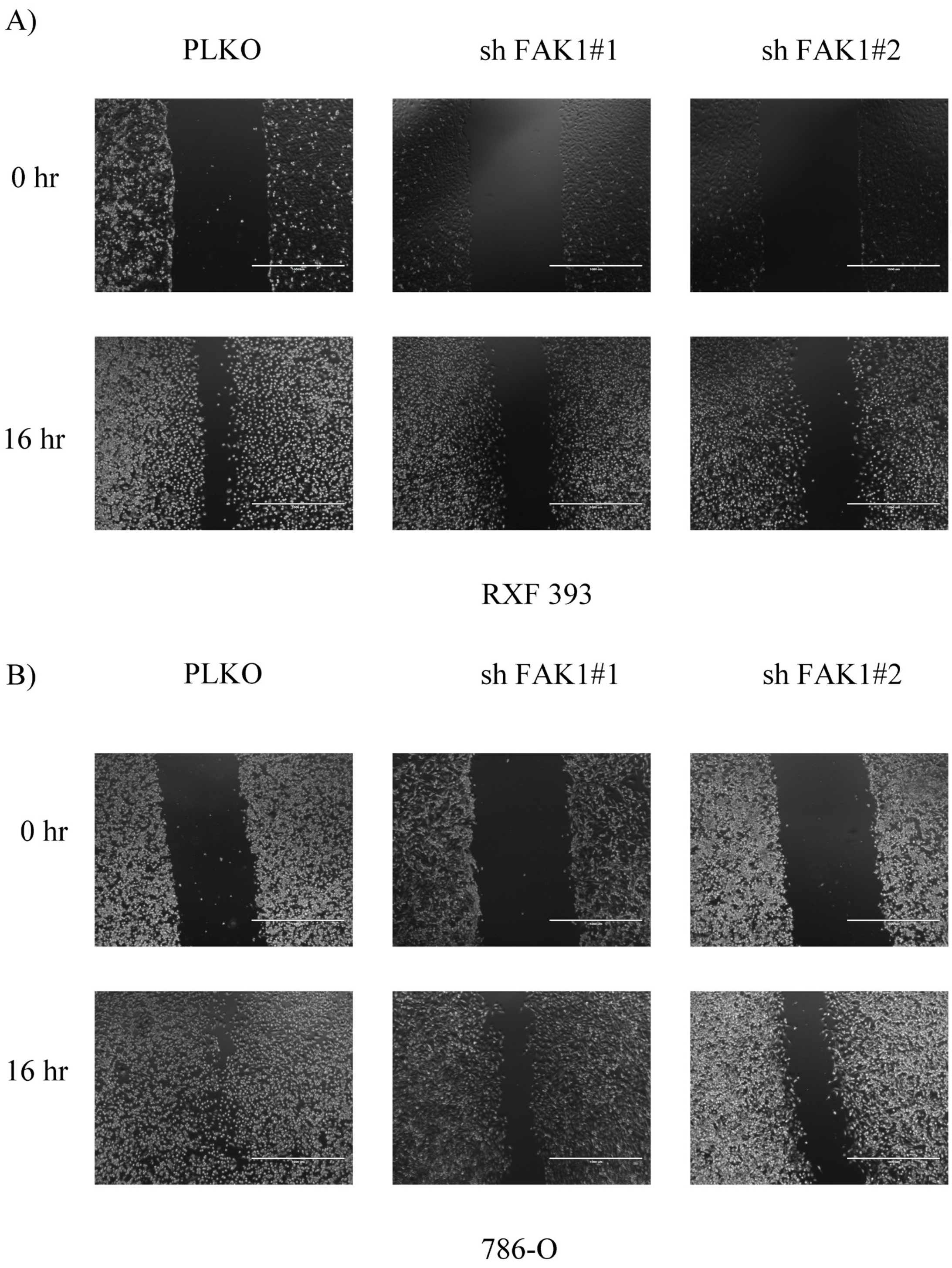

Supplementary Figure 2: FAK1 knockdown decreases migration of RXF393 and 786-O RCC cell lines. **(A)** Migration was assessed in RXF 393 cells transduced with control vector alone or with FAK1 shRNA at 16 h using a wound healing assay and the relative distance of migration was measured. Data was quantified and representative images from 3 replicate experiments are shown. **(B)** Migration was assessed in 786-O cells transduced with control vector alone or with FAK1 shRNA at 16 h using a wound healing assay and the relative distance of migration was measured. Data was quantified and representative images from 3 replicate experiments are shown.

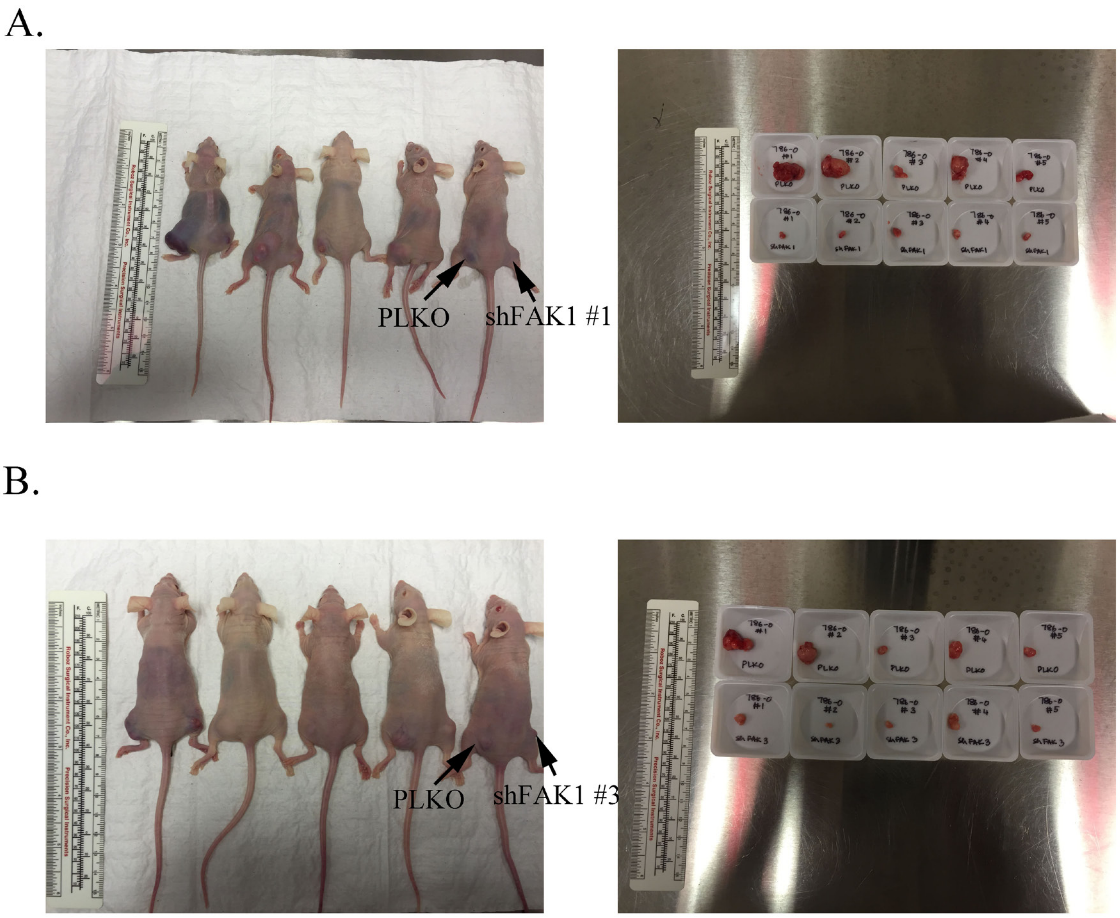

Supplementary Figure 3: FAK1 knockdown in 786-O cells *in vivo*. Control vector transduced 786-O cells were injected into the left flanks of athymic nude mice and FAK1 knockdown cells in the right flanks of the same mice. 5 animals were injected per group and tumors were harvested and measured at 8 weeks post injection. **(A)** 5 mice with PLKO controls injected on the left flank and shFAK#1 cells injected into the right flank. **(B)** 5 mice with PLKO controls injected on the left flank and shFAK#3 cells injected into the right flank.

Supplementary Table 1: Characteristics of tumors analyzed

Parameter	Number
Total number of tumors	96
Total number of tumors eligible for analysis	92[a]
Primary renal tumor	80
Metastatic site	12
% tumor cells (median, range)	90% (<10%-100%)
% necrosis (median, range)	20% (0%-80%)

[a]Four tumors were ineligible for analysis due to poor signal.

Supplementary Table 2: Relative distance measured (in arbitrary units) on wound healing assay in 3 independent ex periments

	GSK2256098	0 h			24h		
		mean	SD	n	mean	SD	n
RXF 393	0µM	1	0.043812	3	0.231325	0.059863	3
	10µM	1	0.027556	3	0.621895	0.021949	3
	20µM	1	0.015616	3	0.549535	0.046118	3
786-O	0µM	1	0.051636	3	0.373002	0.064166	3
	10µM	1	0.009028	3	0.645538	0.031667	3
	20µM	1	0.021049	3	0.606764	0.025629	3

	Cells	0 h			16 h		
		mean	SD	n	mean	SD	n
RXF 393	PLKO	1	0.056792	3	0.19856	0.079863	3
	shFAK1 #1	1	0.05789	3	0.4709	0.022319	3
	shFAK1 #2	1	0.0287	3	0.449535	0.046118	3
786-O	PLKO	1	0.051636	3	0.069	0.094166	3
	shFAK1 #1	1	0.009028	3	0.2489	0.089167	3
	shFAK1 #2	1	0.021049	3	0.306764	0.025629	3
